# Case Report and Literature Review: Bacterial Meningoencephalitis or Not? *Naegleria fowleri* Related Primary Amoebic Meningoencephalitis in China

**DOI:** 10.3389/fped.2022.785735

**Published:** 2022-04-08

**Authors:** Wenjuan Zhou, Yuzhen Ouyang, Di Zhang, Sheng Liao, Hui Liang, Lingling Zhao, Chunyuan Chen

**Affiliations:** ^1^Third Xiangya Hospital, Central South University, Changsha, China; ^2^Xiangya School of Medicine, Central South University, Changsha, China

**Keywords:** *Naegleria fowleri*, primary amoebic meningoencephalitis, pediatrics, metagenomic next-generation sequencing, early diagnosis

## Abstract

In China, a 9-year-old boy was transferred to the hospital with fever, vomiting, and headache. The disease rapidly deteriorated into vague consciousness. Applying conventional clinical examinations such as blood and cerebrospinal fluid (CSF) tests, the diagnosis of bacterial meningoencephalitis was first drawn, and expectant treatments were adopted immediately. However, the symptoms did not alleviate, adversely, this boy died 3 days after admission. Considering the skeptical points of the duration, such as the unknown infectious bacteria and the pathogen invasion path, blood and CSF samples were then sent for metagenomic next-generation sequencing (mNGS) to ascertain the cause of death. The 42,899 and 1,337 specific sequences of *N. fowleri* were detected by mNGS in the CSF sample and the blood sample, respectively. PCR results and pathological smear subsequently confirmed the mNGS detection. The patient was finally diagnosed as primary amoebic meningoencephalitis. Besides, in this article, 15 similar child infection cases in the past 10 years are summarized and analyzed to promote the early diagnosis of this rare disease.

## Introduction

Free-living amoebae have more than 40 genera, but only four were found related to human diseases, including *Acanthamoeba* spp., *Balamuthia mandrillaris, Naegleria fowleri* (*N. fowleri*), and *Sappinia dialoidea* (1). Thereinto, the infection of *N. fowleri* is known to cause an acute and deadly central nervous system disease-primary amoebic meningoencephalitis (PAM), which was first reported in 1965 (2). In the past 55 years, the comprehensive understanding of *N. fowleri* such as its ecology, pathogenesis, epidemiology, and clinical management have been reviewed by numerous researchers (3, 4). However, there still lacks effective diagnosis methods to discriminate PAM from bacterial meningoencephalitis and no drugs are found aimed at this infection (5). Making matters worse, increasing novel cases were reported in more areas worldwide, including Asia (6).

Since 1993, only a few cases have been diagnosed as PAM caused by *N. fowleri* in East Asia (7). In 2011, an *N. fowleri* infection case was reported in Taiwan, and one thermal spring was found contaminated by *N. fowleri* in their later detection (8). In the Chinese mainland, there are three cases suspected as PAM based on amoebic trophozoites in the cerebrospinal fluid (CSF) or brain tissue after death, which is reported in a Chinese journal without an English citation version. However, due to a lack of other potent evidence, a precise diagnosis of *N. fowleri* infection was not put forward. Until August 2016, a 42-year-old male was first diagnosed as PAM with *N. fowleri* infection in the Chinese mainland and unfortunately passed away 14 days after symptoms occurred (9, 10). Later in 2020, reports showed two children (a 2-year-old in Lanzhou and a 13-year-old in shanghai) diagnosed as Granulomatous amoebic encephalitis (GAE) caused by *Balamuthia mandrillaris* (11, 12). According to our retrieval, no other related cases were reported in mainland China. Here, we report the first pediatric *N. fowleri* infection case with an extremely acute disease course in the Chinese mainland. Besides, this study also summarizes 16 *Naegleria* infection cases, including their clinical indicators and diagnosis methods, which can assist doctors in identifying this rare and emerging infection in time.

## Case Description

**Figure 1:**
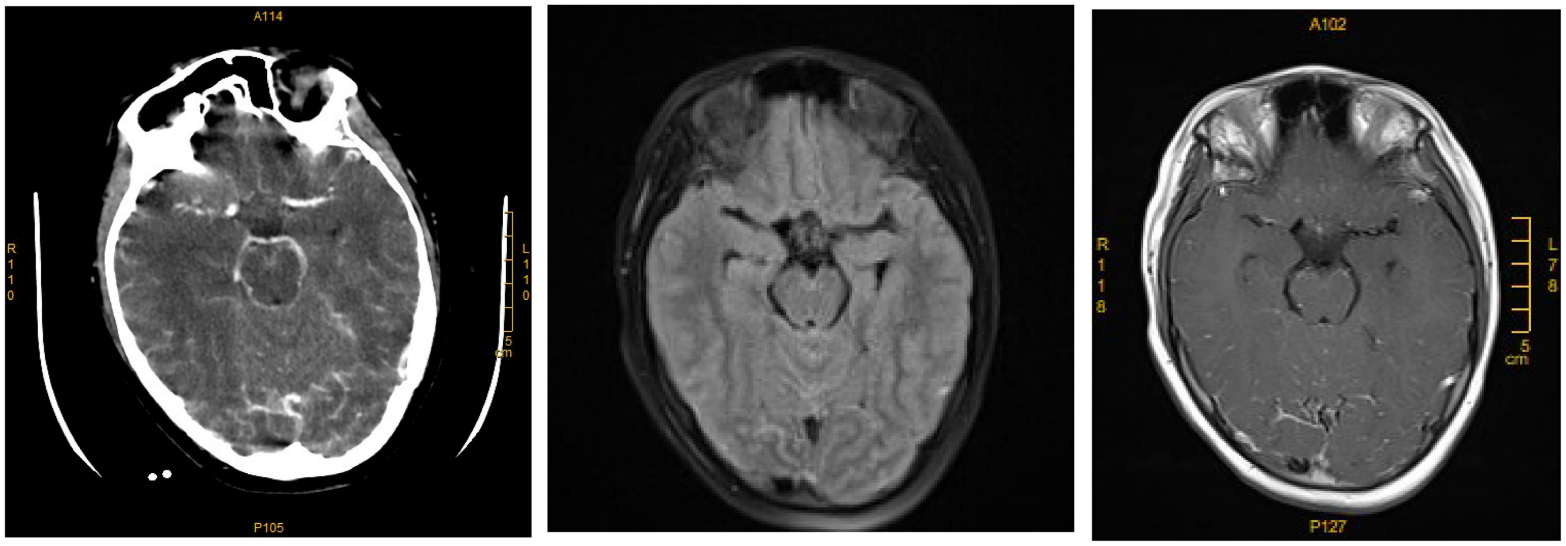
Computed tomography scan and magnetic resonance imaging (MRI) of the primary amoebic meningoencephalitis patient's brain. CT enhancement, T2 flair, and T1 enhancement with leptomeningeal linear enhancement in the occipital lobe.

On August 17, 2020, a 9-year-old boy with fever (T_max_ 40.2°C) was admitted to the Third Xiangya Hospital, Changsha, China. His disease broke out on the 16th with fever and vomiting. Upon admission, the blood test showed leukocytosis of 18.24 × 10^9^/L (92.1% neutrophils). He was treated with Ibuprofen and piperacillin-tazobactam, but the condition did not improve. At 12:00 that day, the child appeared with a headache and vague consciousness and was transferred into the pediatric intensive care unit (PICU) for further treatment immediately. Besides, the child exhibited repetitive high fever, hypertension (149/86 mm Hg), relatively slow heart rate (62/min), positive meningeal irritation signs, and neck stiffness. No positive findings were found in his personal history and contact history of infectious diseases. Therefore, the initial diagnosis was intracranial infection with intracranial hypertension. To ascertain the infection causes, several examinations were adopted, including CSF examination, head computed tomography (CT), and routine blood test. A lumbar puncture discovered that the CSF was purulent in appearance, and the CSF pressure was higher than 300 mmH_2_O. Elevated levels of white blood cells (WBCs 3.25 × 10^9^/L, 70% multi-nucleus), the protein concentration of 2,606 mg/dL, glucose concentration of 0.9 mg/dL in the CSF, and the blood glucose was 127.8 mg/dL at the same time. MRI showed unremarkable T2 flair and T1 enhancement of the boy's brain leptomeningeal linear enhancement in the occipital lobe (Figure 1). The results of routine blood checks, CRP, PCT, and ESR after admission are shown in Supplementary Table S1. Furthermore, hepatic function, renal function, electrolyte results, and respiratory virus examination were all normal.

**Figure 2:**
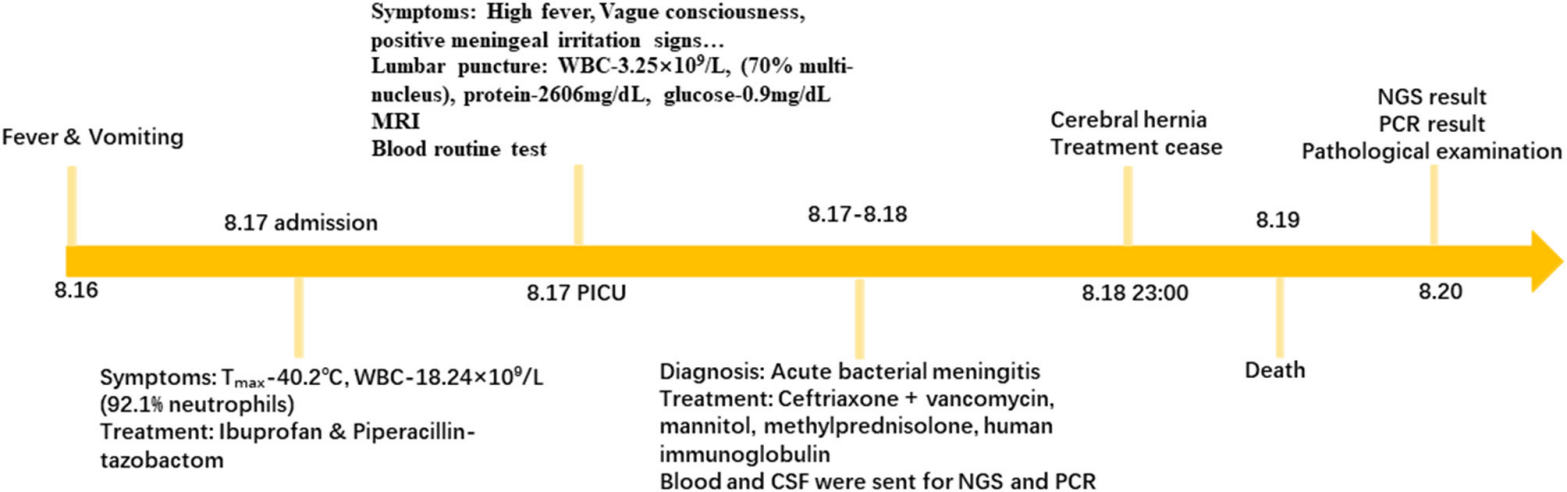
A timeline exhibits the relevant data from the episode of care.

Based on these results and clinical experience, the diagnosis of acute bacterial meningitis was considered. Targeted actions including “Ceftriaxone + vancomycin” anti-infection, “mannitol” to reduce intracranial pressure, “methylprednisolone” for anti-inflammatory purpose, “human immunoglobulin” to improve the body's passive immunity, and limit fluid volume were carried out. However, instead of improvement, at 23:00 on 18th, the child's blood pressure and heart rate suddenly decreased with mydriasis appearance and autonomous respiration arrest. The patient was judged to develop a cerebral hernia. Although the heart rate and blood pressure were stabilized shortly after treatment, the pupillary light reflex and autonomous respiration did not return. The patient's family decided to cease treatment. After removing the life support apparatus, the patient was pronounced dead on 19th. The entire process is briefly exhibited in Figure 2.

## Diagnosis and Outcome

According to the patient's symptoms and examinations, a reasonable diagnosis of “acute bacterial meningitis” could be given. However, the disease progressed quickly and did not respond well to our treatment. Moreover, the CSF and blood culture were all negative. Besides, no significant inflammation in the mastoid, paranasal sinuses, and middle ear were found by both rhinoscopy and CT. There was also no skin infection lesion or skull defect by re-examination. So, the pathogen and its infection pathway remained unknown.

**Figure 3:**
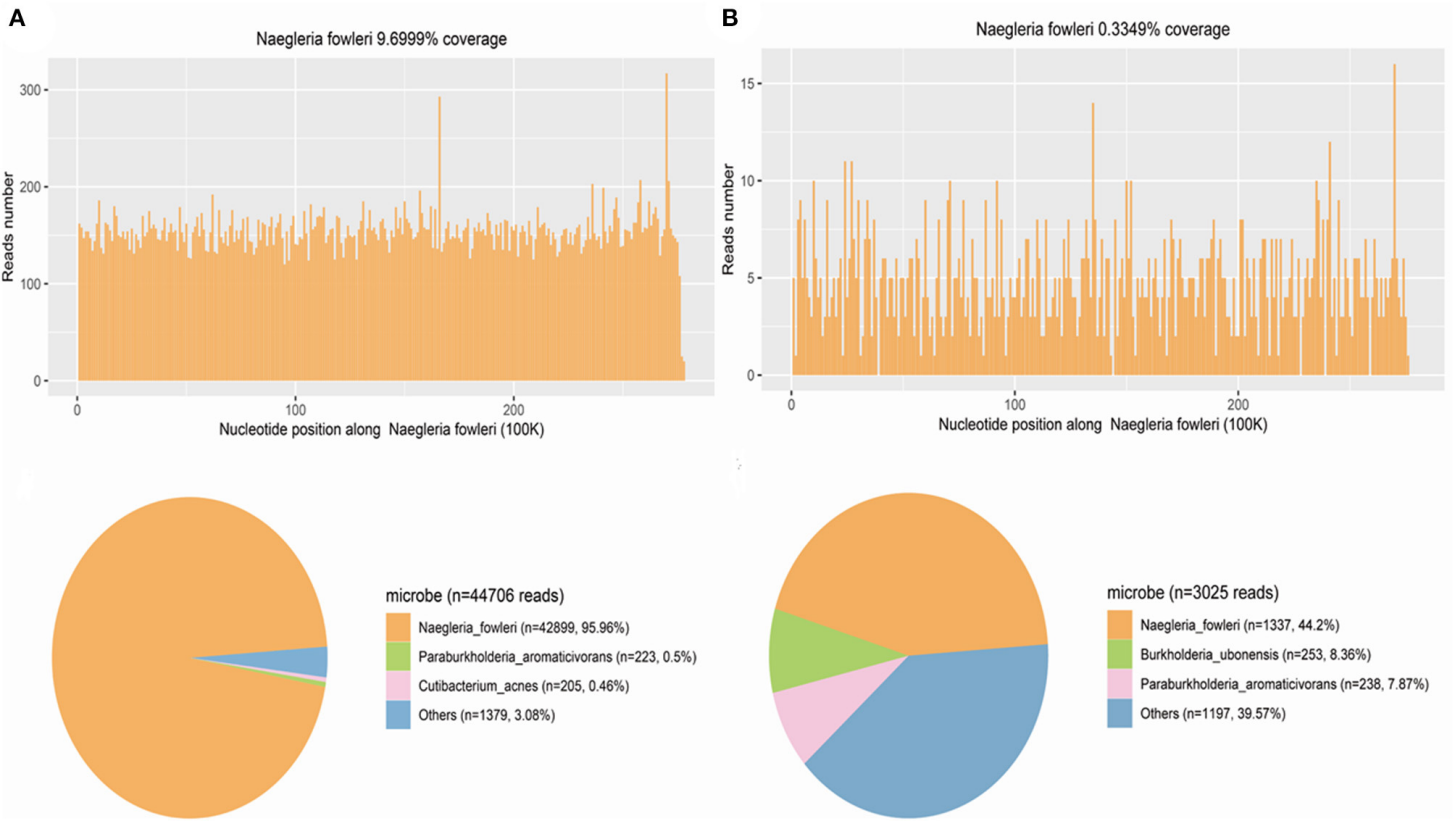
NGS result. **(A)** *N. fowleri* infection diagnosis based on NGS on CSF sample. The upper panel is the *N. fowleri* reads in CSF on the genome mapping, whereas the distribution of the reads of microbes and unclassified reads without human host reads. The number of *N. fowleri* specific sequences is 42,899, which occupies 95.96% of all microbe sequences, with high confidence of *N. fowleri* infection. **(B)** NGS on serum sample for *N. fowleri* infection diagnosis. The *N. fowleri* reads in serum on the genome mapping are on the upper panel, whereas the reads distribution of microbes and unknown sequences in the absence of human host reads. There are 3,025 *N. fowleri* specific sequences that take up 44.2%of all microbe sequences, in high confidence of *N. fowleri* infection.

**Figure 4:**
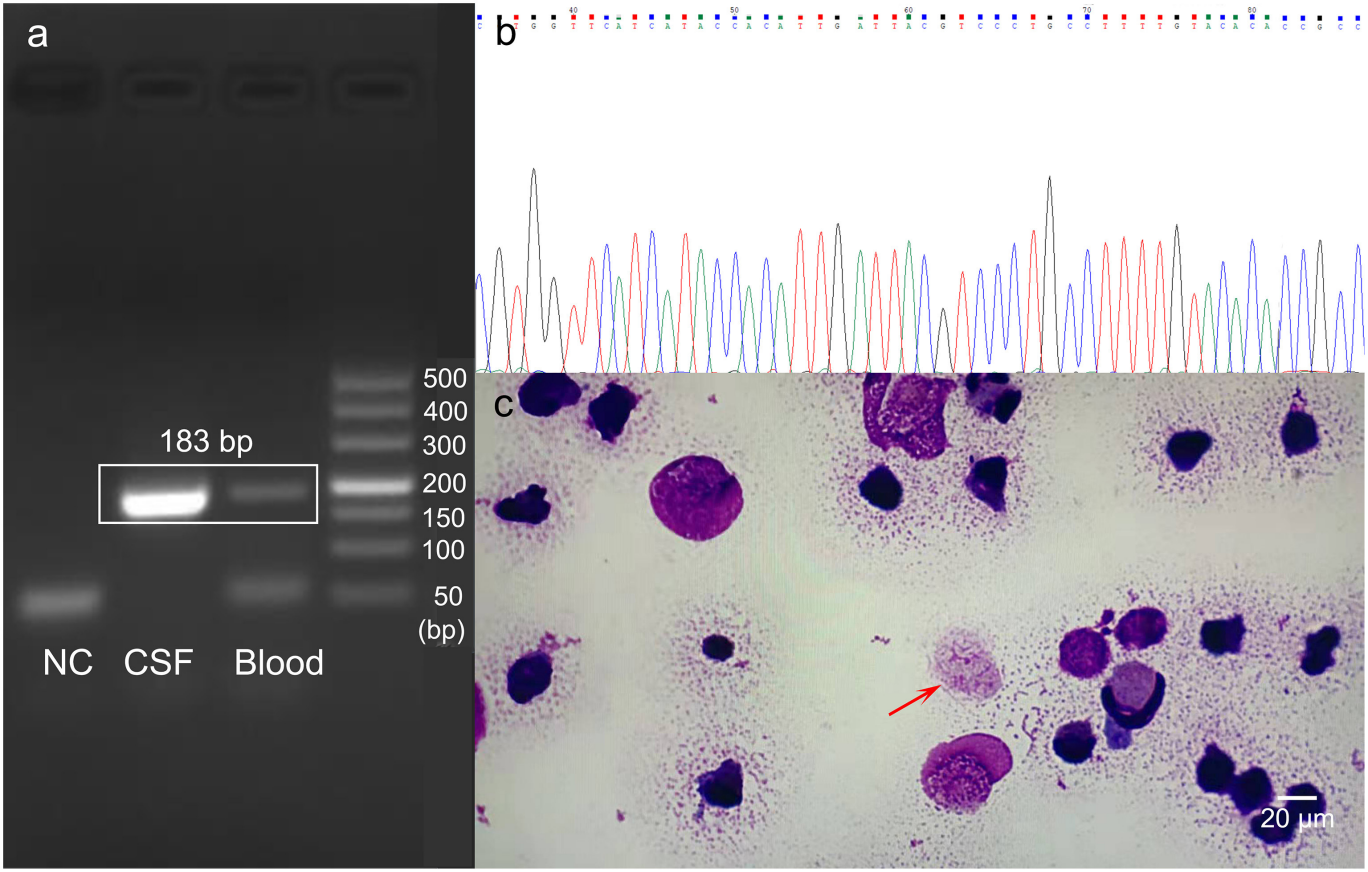
*N. fowleri* infection verification by PCR and pathological examination of CSF slides. **(a)** PCR amplification bands exhibited by agarose gel electrophoresis. NC: negative control; marker: DL500. **(b)** The PCR result of *N. fowleri* in this patient detected by Sanger sequencing. **(c)** Image of *N. fowleri* on Wright-Giemsa-stained CSF slides (1,000x, oil immersion). The arrow indicates *N. fowleri* trophozoites (Scale bar: 20 μm).

The CSF and blood samples were sent for further detection using metagenomic next-generation sequencing (mNGS) in Hugo-Biotech Company, Beijing. The DNA was extracted and purified from 200 uL CSF supernatant and 200 uL blood plasma according to the manufacturer's instruction of TIANGEN kit with DNA library constructed using QIAseq^TM^ Ultra low input library kit. Qualified libraries with different barcode labeling were pooled together and then NGS was conducted on the Illumina Nextseq platform. In the CSF and blood sample, when the human genome was excluded, the NGS identified 42,899 (out of 44,706) and 1,337 (out of 3025) specific sequence reads belonging to *N. fowleri* genome, respectively (Figure 3). As *N. fowleri* DNA sequences occupied 95.96 and 44.2% of the whole microbe genome in the CSF and blood sample, it was reasonable to consider PAM as the diagnosis. To verify the results, PCR detection of *N. fowleri* and Sanger sequencing were adopted. Using the specific primers CAAACACCGTTATGACAGGG and CTGGTTTCCCTCACCTTACG covering ribosomal 5.8S ribosomal RNA gene and internal transcribed spacer 2 gene, the electrophoresis result indicated the specific band was about 183 bp, which was the positive band of *N. fowleri* infection in the CSF and blood sample. Besides, the brightness of the PCR amplification band was consistent with the difference in the number of reads detected by NGS (Figure 4a). As for the NCBI blast, the nucleotide sequence in 183 bp showed 99% homology with the reference sequence, indicating *N. fowleri* infection in high confidence (Figure 4b). Moreover, dead amoebae were also found by microscopic observation after smearing the CSF through Wright-Giemsa-stained CSF slides (Figure 4c). In addition, patient history collected from his family showed that the child went to a public swimming pool for recreation and was choked 6 days before the disease occurred. Then, the child was deemed to have sinusitis, but an ear-nose-throat (ENT) examination showed no evidence. Furthermore, the fishy and odorous secretions in the oral and nasal cavity were found during intubation, but unfortunately these secretions were not sent for pathological examination the first time. The patient was finally diagnosed as primary amoebic meningoencephalitis by *N. fowleri*.

## Discussion

In this study, we reported the first pediatric *N. fowleri* infection case in the Chinese mainland. The child was misdiagnosed as “acute bacterial meningoencephalitis” until the NGS results came out. Patients with *N. fowleri* infection also experienced a 1–7 day disease latency period, and then initial symptoms occurred such as fever, headache, and vomiting, which is not distinctive from bacterial meningitis (13). The misdiagnosis as “bacterial meningitis” frequently happens, which is one of the factors leading to its high mortality. Therefore, we summarize and discuss how to differentiate *N. fowleri* related primary amoebic meningoencephalitis from bacterial meningoencephalitis, which may help the early diagnosis.

### Differences of Meningoencephalitis/Meningitis Caused by Bacteria and *N. fowleri*

The first point that needs attention is that purulent meningoencephalitis/meningitis is not equal to bacterial meningoencephalitis/meningitis. Purulent meningoencephalitis/meningitis can also be caused by *N. fowleri* infection as our and previous cases reported (14, 15). Moreover, the suspect diagnosis of bacterial meningitis should be put forward when purulent meningitis or meningoencephalitis are found, but no bacterial is discovered in the CSF (16).

Furthermore, in bacterial meningitis, the bacteria usually reach the meninges by any of the following mechanisms: (1) hematogenous spread from systemic infection foci, (2) spread from nearby infected mastoid, middle ear, or paranasal sinuses, (3) through the choroid plexus, (4) the superficial parenchymal abscess flowing into the subarachnoid space, and (5) penetrating trauma on the skull (17). If the infection pathway cannot be determined, the diagnosis of “acute bacterial meningitis” should be suspected.

### Clinical Features of Meningoencephalitis by *N. fowleri* Infection

To make an accurate diagnosis as soon as possible, we summarized the clinical features of this case and another 15 cases of children infected with PAM worldwide in the past 10 years (Supplementary Table S2) (13, 18–26). Among these 16 cases, 12 of them were reported in the United States, indicating its high incidence there. As for the initial symptom, symptoms of increased intracranial pressure such as headache, fever, and vomiting were common. Notably, the appearance of lethargy, seizures, altered mental status, photophobia, and nausea were also worthy of attention. Moreover, in CSF examination, most cases showed an increase of WBC (from 130/mm^3^ to 15,406/mm^3^) with neutrophil predominance, even six cases reported the appearance of RBC. Eight cases exhibited decreased CSF glucose level (≤40 mg/dL) and the CSF protein increased in 14 cases (out of 16) except two cases which did not mention the protein index. In addition, not displayed in the table, the main CT feature was edema including diffuse edema and focal edema. It was worth noting that in some cases CT results showed no obvious abnormality or mild manifestation at first, but manifested in advanced stages.

Thus, when an acute headache, fever, and vomiting occur, with increased WBC amount (neutrophil predominance) and protein concentration, and decreased glucose level, meningoencephalitis due to *N. fowleri* infection should be considered, especially when the CSF is purulent but the CSF culture is negative.

### The Diagnosis of *N. fowleri* Caused Meningoencephalitis

The common detection methods of *N. fowleri* include indirect immunofluorescence, PCR, CSF wet mount, and mNGS. Among these 16 patients, nine were diagnosed after death, while 6 patients were diagnosed as PAM within hospital days. Two patients successfully survived due to the early diagnosis and timely treatment with satisfactory recovery. CSF wet mount is the most immediate and fast method and is commonly used for a diagnosis before death. PCR testing, brain tissue immunofluorescent analysis (IF), and NGS were also applied but were more time-consuming. If the brain crest fluid looks purulent, do a wet mount test and smear as soon as possible while culturing. It is important that, if there are many doubts about the diagnosis of bacterial meningitis, PCR or NGS should be performed immediately to assist the diagnosis. We expect the improvement of NGS detection speed in the future to help doctors make a faster and more accurate diagnosis.

In summary, we illustrated the first case of child *N. fowleri* infection in China, which had rapid progress and high mortality. After reviewing pediatric PAMs worldwide, some clues and methods were found promising to diagnose the disease.

## Data Availability Statement

The original contributions presented in the study are included in the article/Supplementary Material, further inquiries can be directed to the corresponding author/s.

## Ethics Statement

The studies involving human participants were reviewed and approved by the Ethical Review Committee of Third Xiangya Hospital. Written informed consent to participate in this study was provided by the participants' legal guardian/next of kin. Written informed consent was obtained from the individual(s), and minor(s)' legal guardian/next of kin, for the publication of any potentially identifiable images or data included in this article.

## Author Contributions

WZ and YO designed and wrote the report. SL and HL participated in drafting and revising the manuscript. CC and LZ reviewed the manuscript for its intellectual content and revised the entire work. All authors approved the final manuscript as submitted and agreed to be accountable for all aspects of the work.

## Funding

This study was funded by the Project for Xiangya famous doctor in Central South University (2014 [68]).

## Conflict of Interest

The authors declare that the research was conducted in the absence of any commercial or financial relationships that could be construed as a potential conflict of interest.

## Publisher's Note

All claims expressed in this article are solely those of the authors and do not necessarily represent those of their affiliated organizations, or those of the publisher, the editors and the reviewers. Any product that may be evaluated in this article, or claim that may be made by its manufacturer, is not guaranteed or endorsed by the publisher.

## Abbreviations

*N. fowleri*: *Naegleria fowleri*
PAM: primary amoebic meningoencephalitis
CSF: cerebrospinal fluid
CRP: c-reactive protein
WBC: white blood cell
PCT: procalcitonin
ESR: erythrocyte sedimentation rate
NGS: next generation sequencing
ENT: ear-nose-throat
IF: immunofluorescent analysis.
