# Supplementary material for: Case Report and Literature Review: Bacterial Meningoencephalitis or Not? Naegleria fowleri Related Primary Amoebic Meningoencephalitis in China
Source: Front Pediatr. 2022 Apr 8;10:785735. doi: 10.3389/fped.2022.785735 (PMC9033202; doi:10.3389/fped.2022.785735)
Supplement: Supplementary file 1 [file Table_1.DOCX]

| Supplement Table 1: Blood test result | | | | | | | | |
| --- | --- | --- | --- | --- | --- | --- | --- | --- |
| Time | Blood routine examination | | | | C-reactive protein  (mg/L) | | Procalcitonin  (ng/ml) | Erythrocyte Sedimentation rate  (mm/hr) |
|  | Leukocyte  (*10^9/L) | Platelet  (*10^9/L) | Neutrophil Granulocyte Percentage  (%) | Hemoglobin  （g/L） |  |  |  |  |
| 8.17 08:04 | 18.24 | 357 | 92.1 | 135 | | 34.56 | / | / |
| 8.17 18:58 | 21.68 | 274 | 86.5 | 120 | | 74.26 | 1.1370 | / |
| 8.18 08:39 | 15.96 | 244 | 88.9 | 128 | | 164.68 | 2.4339 | 88 |
| 8.19 11:41 | 12.12 | 224 | 92.4 | 133 | | 172.48 | 2.8480 | / |
