# Supplementary material for: Case Report and Literature Review: Bacterial Meningoencephalitis or Not? Naegleria fowleri Related Primary Amoebic Meningoencephalitis in China
Source: Front Pediatr. 2022 Apr 8;10:785735. doi: 10.3389/fped.2022.785735 (PMC9033202; doi:10.3389/fped.2022.785735)
Supplement: Supplementary file 2 [file Table_2.docx]

| Patient | Country | Initial symptoms | CSF | Diagnosis method | Outcome | Ref |
| --- | --- | --- | --- | --- | --- | --- |
| 1 | USA | Fever, Headache, Neck stiffness, Photophobia, Vomiting | WBC: 985 cells/mm^3^(Neutrophils 79 %); Protein: 79 mg/dL; Glucose: 69 mg/dL | PCR,CNS Autopsy,Immunofluorescencestaining | Died (After) | 13 |
| 2 | India | Fever, Vomiting, Seizures, Decreased urine output | WBC: 1020 cells/mm^3^; Protein:363mg/dL; Glucose:54mg/dL | PCR,CSF wet mount | NM (Before) | 18 |
| 3 | USA | Fever, Headache, Lethargy  Altered mental status | WBC: 3808 cells/mm^3^ (Neutrophils 78%), Protein: 410 mg/dL; Glucose: <10mg/dL | PCR | Died (After) | 19 |
| 4 | Bangladesh | Fever, Headache, Vomiting, Neck stiffness | WBC: 2000 cells; Protein:70 mg/dl; Glucose: 52 mg/dl | PCR | Died (After) | 20 |
| 5 | USA | Fever, Headaches, Vomiting, Altered mental status, seizure, Ambulating difficulty, Neck stiffness | WBC: 2078/mm^3^(Neutrophils 37%); Protein: 862 mg/dL; Glucose: < 20 mg/dL | PCR,CSF wet mount | Died (Before) | 21 |
| 6 | USA | Headache, Fever, Muscle weakness | WBC: 2200/mm^3^ (Neutrophils: 89%); Protein: 1233 mg/dL; Glucose: < 20mg/dL | PCR,CSF wet mount | Died (Before) | 21 |
| 7 | USA | Headache, Vomiting, Fever, Neck stiffness, general malaise | WBC: 7950 cells/mm^3^; Protein: 622 mg/dL; Glucose: 1 mg/dL; RBC:40cells/mm^3^ | Brain tissue IIF | Died (After) | 22 |
| 8 | USA | Headache, Vomiting,Fever, Neck stiffness and pain, Altered mental status, Glasgow coma scale 15, nonfocal neurological examination findings | WBC: 4,180 cells/mm^3^; Protein: 362 mg/dL; Glucose: 24 mg/dL; RBC: 220 cells/mm^3^ | PCR,CSF wet mount | Died (Before) | 22 |
| 9 | USA | Headache, Fever, Alteredmental status, Neck stiffness, Nausea, Photophobia | WBC: 1,000 cells/mm^3^; Protein:151 mg/dL; Glucose: 46 mg/dL; RBC: 200 cells/mm^3^ | PCR,Brain tissue IIF | Died (After) | 22 |
| 10 | USA | Headache, Vomiting, Fever, Lethargy, Abdominal pain | WBC: 10,113 cells/ mm^3^; Protein: 390 mg/dL; Glucose: 8 mg/dL; RBC: 27 cells/ mm^3^ | PCR,CSF wet mount | Died(After) | 22 |
| 11 | USA | Headache, Vomiting, Fever, Lethargy, Confusion, Blurred vision, Diplopia | WBC: 1,463 cells/ mm^3^; Protein: 411 mg/dL; Glucose: 68 mg/dL; RBC: 228 cells/ mm3 | PCR,CSF wet mount | Died (Before) | 22 |
| 12 | USA | Headache, Abdominal pain, Neck soreness | WBC: 8150 cells/ mm^3^ (90% neutrophils); Protein: 461 mg/dL; Glucose level: < 20 mg/dL; RBC: 800 cells/ mm^3^ | Histopathology of CSF and brain tissue | Died (After) | 23 |
| 13 | USA | Headache, Fever, Vomiting, Akinesia | NM | CSF wet mount | Survived (Before) | 24 |
| 14 | USA | Headache, Vomiting  Diarrhea | WBC: 1139 cells/mm^3^ (neutrophilic predominance); Protein: 172 mg/dL | Histopathology of brain tissue,CSF wet mount | Died (After) | 25 |
| 15 | India | Headache, Fever, Altered sensorium | Cell count of 415 cells/mm^3^ and predominance of neutrophils | CSF wet mount | Survived (Before) | 26 |
| 16(ours) | China | Fever, Vomiting, Headache, Altered mental status | WBC: 3250 cells/mm^3^ (70% neutrophils); Protein: 2602mg/dl; Glucose: 0.9 mg/dL | NGS,PCR,CSF wet mount | Died (After) |  |

*NM: not mentioned;IIF: Indirect immunofluorescence specific for N.fowleri
